# Femur Auxetic Meta-Implants with Tuned Micromotion Distribution

**DOI:** 10.3390/ma14010114

**Published:** 2020-12-29

**Authors:** Naeim Ghavidelnia, Mahdi Bodaghi, Reza Hedayati

**Affiliations:** 1 Department of Mechanical Engineering, Amiabir University of Technology (Tehran Polytechnic), Hafez Ave, Tehran 1591634311, Iran; n.ghavidelnia@aut.ac.ir; 2 Department of Engineering, School of Science and Technology, Nottingham Trent University, Nottingham NG11 8NS, UK; mahdi.bodaghi@ntu.ac.uk; 3 Novel Aerospace Materials Group, Faculty of Aerospace Engineering, Delft University of Technology (TU Delft), Kluyverweg 1, 2629 HS Delft, The Netherlands

**Keywords:** 3D auxetic, hip implant, mechanical metamaterial, porous biomaterials

## Abstract

Stress shielding and micromotions are the most significant problems occurring at the bone-implants interface due to a mismatch of their mechanical properties. Mechanical 3D metamaterials, with their exceptional behaviour and characteristics, can provide an opportunity to solve the mismatch of mechanical properties between the bone and implant. In this study, a new porous femoral hip meta-implant with graded Poisson’s ratio distribution was introduced and its results were compared to three other femoral hip implants (one solid implant, and two porous meta-implants, one with positive and the other with a negative distribution of Poisson’s ratio) in terms of stress and micromotion distributions. For this aim, first, a well-known auxetic 3D re-entrant structure was studied analytically, and precise closed-form analytical relationships for its elastic modulus and Poisson’s ratio were derived. The results of the analytical solution for mechanical properties of the 3D re-entrant structure presented great improvements in comparison to previous analytical studies on the structure. Moreover, the implementation of the re-entrant structure in the hip implant provided very smooth results for stress and strain distributions in the lattice meta-implants and could solve the stress shielding problem which occurred in the solid implant. The lattice meta-implant based on the graded unit cell distribution presented smoother stress-strain distribution in comparison with the other lattice meta-implants. Moreover, the graded lattice meta-implant gave minimum areas of local stress and local strain concentration at the contact region of the implants with the internal bone surfaces. Among all the cases, the graded meta-implant also gave micromotion levels which are the closest to values reported to be desirable for bone growth (40 µm).

## 1. Introduction

There are two established methods for femoral implant fixation namely cementation [1] and press-fit [2]. The cementless implants have the advantage of providing the possibility of bone ingrowth inside porous implants or porous surfaces of solid implants. Completely porous implants usually have lower stiffness levels when compared to solid implants thus allowing for a better transfer of load from implant to bone and hence decreases the stress shielding phenomenon which can lead to bone resorption. Initial and long-term stability of the femoral implant is highly dependent on the relative motion of the implant with respect to the bone in its vicinity (known as micromotion, micro-movement, or relative motion [3]) and is of great importance for the long term success of the implant [4]. Due to their higher vulnerability to micromotions, the frequency of aseptic loosening is still higher in cementless femoral stems as compared to cemented types [5].

Some recent studies [6] have shown that an initial press-fit condition in the range of 0.01 mm and 0.1 mm gives the optimal results for stability, durability, and bone growth. Depending on their extent, micromotions can have two opposite effects on bone ingrowth inside the implant. Micromotion less than a critical value (30 µm [7], 40 µm [8], 50 µm [9], 90 µm [10], 100 µm [11]) enhances osseointegration while micromotions larger than 150 µm [8] results in the formation of fibrous tissue in the external surface of the implant. Fibrous tissues are the result of bone tissue repair mechanisms in response to repetitive loading of the implant [12]. Moreover, excessive micromotions can activate osteoclast cells which leads to bone resorption and thus implant failure [13,14]. The first study on the effect of micromotion on the bone ingrowth in porous implants was carried out by Cameron et al. [15,16] in which they showed that bone growth is disturbed by micromotions. The amount of micromotion is significantly affected by the direction of the applied load. Cristofolini et al. [17] showed that micromotions usually cause a gap opening in the medial region and shear slippage in the posterior region.

One of the promising methods to reduce micromotion can be to manipulate Poisson’s ratio of an implant in different locations and directions. Depending on the extent of the load an implant receives and the extent of required in-situ relative lateral movement, an implant can be designed with the desired distribution of lateral movement. This can be accomplished not by normal materials, but by materials that show exotic properties, commonly known as metamaterials. Metamaterials are designer materials that exhibit properties not usually found in nature [18]. Recent advances in additive manufacturing have made it possible to have control over the micro- and macro-structural design features of porous structures [19,20,21,22,23,24,25,26] and manufacture several types of mechanical metamaterials such as Pentamodes [23,27,28,29], auxetics [30,31], and materials with negative compressibility [32]. One of the most commonly known mechanical metamaterials is auxetics that show negative Poisson’s ratio behaviours [33,34]. It means that unlike normal material which expands laterally under compressive loading, they shrink laterally. 

Designing a structure with a wide variety of positive and negative Poisson’s ratio at different locations can lead to implants which have lateral displacements in compliance with the surrounding bone. The most famous and applicable bio-metamaterial structures for this purpose are 3D re-entrant structures which could provide positive or negative values for their Poisson’s ratio depending on their internal struts angles [35]. Such structures due to their wide range of geometrical parameters (such as strut thickness, two types of internal angles, etc.) can also be suitable candidates for designing porous implants with appropriate mechanical properties. The behaviours and mechanical properties of the noted structure have been analyzed analytically and numerically in the literature in several works [36,37,38]. The availability of exact analytical relationships for this structure can help to design and optimize the well-matched structure of porous implants.

In recent years, many researchers have focused on the effects of geometry, porosity, material, micro-architecture, loading, coating, etc., on the micromotion extents in implants especially total hip replacements (THRs). Chen et al. [39] evaluated two types of the femoral stem (anatomic and straight) and investigated their micromotions relative to the femur using finite element (FE) analysis. In another similar study, Bieger et al. [40] studied the primary stability and stress-shielding effect of some stem prostheses namely Fitmore, CLS, and Mayo. Camine et al. [5] developed a µ-CT-based technique to measure gap and micromotion during compressive loading at all exterior locations of the femoral stem. Wang et al. [41] examined a fully three-dimensional optimally graded lattice hip implant to minimize bone resorption and bone–implant micromotion. In a recent study, Kolken et al. [42] presented conceptual hybrid meta-biomaterials with a combination of negative and positive Poisson’s ratio structures. Their results demonstrated improvement in implant–bone contact micromotion under axial loading condition. Kolken et al. [42] implemented well-known re-entrant and honeycomb structures for generating implant hybrid structures in order to minimize the chance of wear and to improve implant fixation at both sides of the hybrid implant faces. 

The goal of this study is to tune the micromotion of hip implants to desirable ranges by tuning the micro-structural features of meta-implants. Gradient distribution of 3D re-entrant unit cells can be a good solution to mitigate micromotions and to improve stress shielding in the hip implant. For this aim, first, analytical relationships for mechanical properties of 3D re-entrant unit cell are derived and then four types of implants (solid implant; meta-implant with positive Poisson’s ratio; meta-implant with negative Poisson’s ratio; and graded meta-implant) are designed and studied using finite element analysis (FEA). As the porous implants are constructed by lattices of metamaterials (auxetics), they are referred to as meta-implants in the rest of the paper. The implants are then designed based on the anatomy of the femur bone to have better initial adjustment and fixation for hip implants. A geometrical model of the femur bone is created precisely by considering both the cancellous and cortical bones to obtain accurate results. Finally, the stress and strain distributions of the implants, as well as the micromotions at bone-implant interfaces (considering both gap and sliding distances), are analysed and compared between the four designs.

## 2. Materials and Methods

In the first part of this section, analytical relationships for the main mechanical properties of 3D re-entrant unit cell, namely elastic modulus and Poisson’s ratio, are derived. The newly developed analytical relationships are more precise when compared to existing relationships available in the literature [35,38]. In the second part of this section, the detailed procedure of numerical modelling and analysis of solid and porous femoral hip meta-implants is described. The details of implementing the derived analytical relationships into creating meta-implants with positive, negative, and graded Poisson’s ratios are also presented.

### 2.1. Analytical Analysis

#### 2.1.1. Stiffness Matrix Derivation

**Figure 1:**
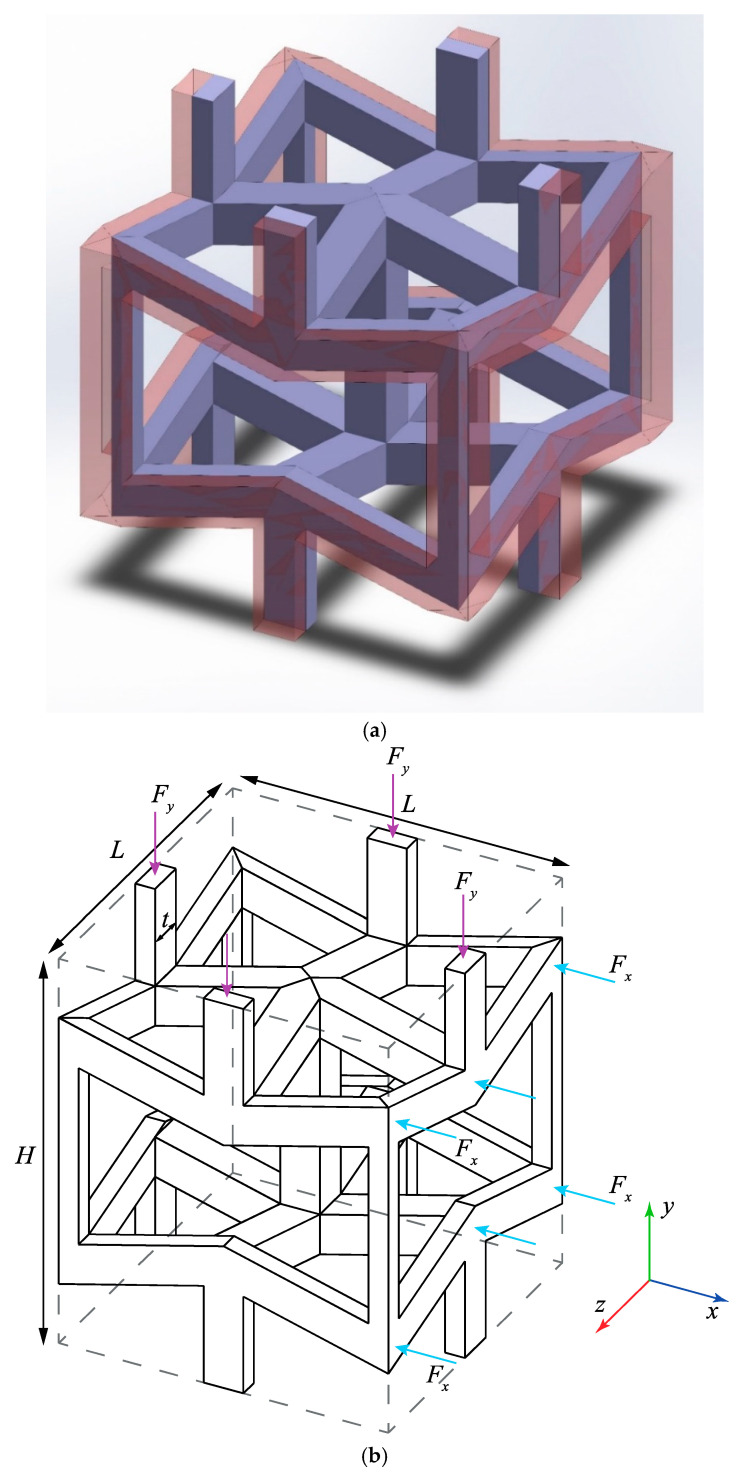
(**a**) 3D re-entrant unit cell (shared struts are highlighted). (**b**) Loading conditions and geometrical parameters of the 3D re-entrant unit cell. (H—length of the unit cell in the y direction, L—length of the unit cell in the x or z directions, F—applied load on the unit cell in the y or x directions, t—thickness of struts of the unit cell).

In this subsection, analytical relationships for elastic modulus and Poisson’s ratio of the re-entrant unit cell are derived as functions of elastic properties of the constituent material ($E_{s}$ and $v_{s}$) and the geometrical parameters of the unit cell. A 3D re-entrant unit cell which is considered for obtaining analytical solutions is shown in Figure 1a. The parts of the neighbour unit cells which share the same struts with the considered unit cells are distinguishable by their light red colour in this figure. The approach of deriving the analytical relationships for the 3D re-entrant structure is the linear superposition method which has been introduced and implemented for solving other structures such as rhombic dodecahedron [43], truncated cube [44], octahedral [45], rhombicuboctahedron [46], etc. To implement the superposition method for obtaining analytical solutions for the 3D re-entrant structure, firstly a definition is needed for the system of equations for the structure in the matrix form of $\left\{F\right\}\;=\;\left[K\right]\left\{q\right\}$. In this equation, $\left\{F\right\}$ is the force vector containing the external forces acting on the structure, $\left[K\right]$ is the stiffness matrix of the system, and the $\left\{q\right\}$ is the displacement vector of the different joints of the unit cell. Since the analytical relationships (say mechanical properties) for the structure are different in the global x and y directions, we will consider two different types of loading conditions to obtain the mechanical properties in these two distinct directions, see Figure 1b. 

**Figure 2:**
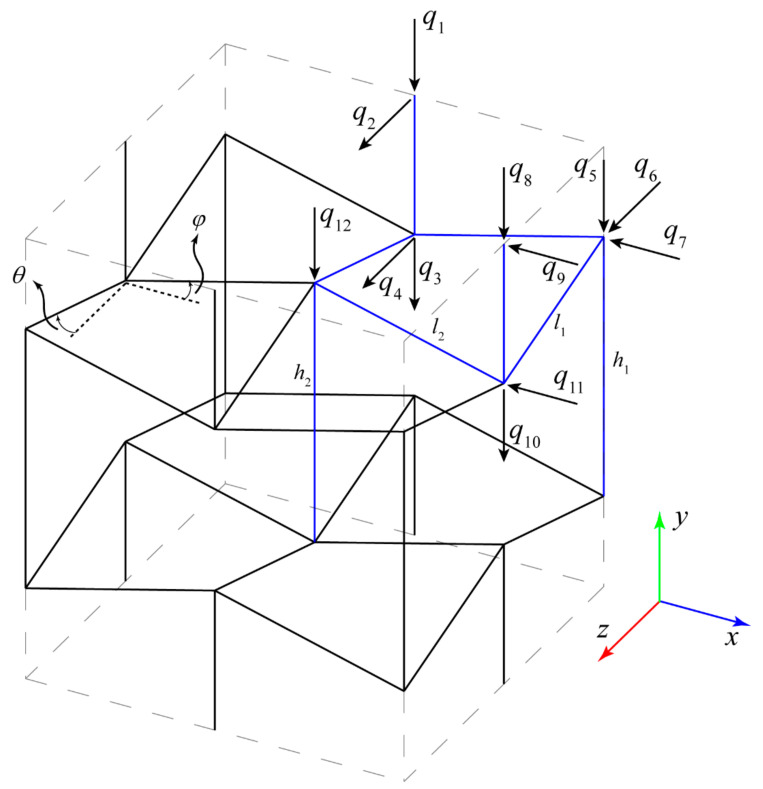
Considered degrees of freedom $q_{i}$ for analytical solution of the structure. The unit cell has four vertical (xy, yz, and two bisectors of xy and yz) and one horizontal (xz) symmetry planes. $h_{1}$, $h_{2}$, and l are dimensional parameters of the unit cell.

**Figure 3:**
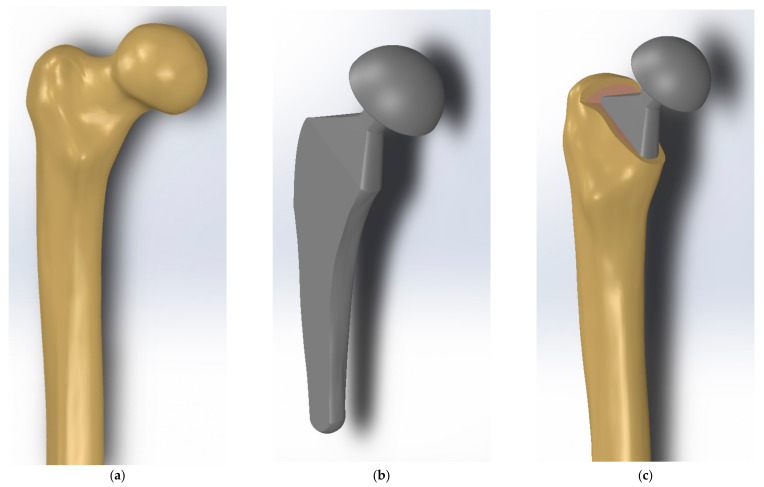
(**a**) Femur 3D CAD model (**b**) Designed Implant 3D model, (**c**) Designed implant assembled into the cortical and trabecular bone.

According to the four vertical (xy, yz, and two bisectors of xy and yz) and one horizontal (xz) symmetry planes of the re-entrant structure and also two considered loading conditions in the x and y directions, the behaviour of the re-entrant unit cell can be modelled completely by 12 distinct degrees of freedom (DOF), see Figure 2. The stiffness matrix $\left[K\right]$ will then be a 12 × 12 symmetrical matrix. The details of calculations for elements of the stiffness matrix can be found in the Supplementary material. To calculate the mechanical properties in two directions, it is necessary to solve the system of equations for two distinct loading conditions such that $\left\{F\right\}=\left\{F_{x}\right\}$ or $\left\{F\right\}=\left\{F_{y}\right\}$. It is worth noting that since we tried to consider a very general condition for the 3D re-entrant structure, each unit cell consists of four strut types with lengths ($l_{1},\;l_{2}\;,\;h_{1},h_{2}$) and three types of cross-sectional areas ($A_{1},\;A_{2},\;A_{3}$) after excluding the shared parts of neighbours struts see Figure 3. The unit cell is fully defined with two unique angles θ and φ and lengths L and H. Angle θ is defined as the angle between strut $l_{1}$ and the horizontal plane and angle φ is defined as the angle between strut $l_{2}$ and the horizontal plane (Figure 2). By considering the total occupied volume of the re-entrant unit cell ($V_{total}=HL^{2}$) and strut thickness t for square cross-section as well as the above-mentioned geometrical assumptions, the relative density of 3D re-entrant unit cell could be obtained readily as:

(1) $$\mu =\frac{V_{struts}}{V_{total}}=\frac{4A_{2}h_{1}+4A_{1}h_{1}+A_{3}h_{2}+16A_{2}l_{1}+8A_{3}l_{2}}{HL^{2}}=\frac{3t^{2}h_{1}+t^{2}h_{2}+8t^{2}l_{1}+8t^{2}l_{2}}{HL^{2}}$$

where $A_{1},\;A_{2}$ and$\;A_{3}$ are:

(2) $$A_{1}=t^{2}/4,\;A_{2}=t^{2}/2\;,\;A_{3}=t^{2}$$

To obtain the elements of ith column of the stiffness matrix $\left[K\right]$, we displace DOF i while keeping the other DOFs fixed. By calculating the of forces that should be applied to each DOF to create the above-mentioned displacement, the elements of the ith column of the stiffness matrix could be obtained. Two well-known beam theories, namely Euler-Bernoulli and Timoshenko theories, will be implemented for analytical analysis of the 3D re-entrant unit cell. The parameters $S_{i}$ and $T_{i}$ have been used for summarizing the relationships. The term $S_{i}$ denotes the axial tension/compression term which is identical for both Euler-Bernoulli and Timoshenko theories and it is defined as:

(3) $$S_{i}=\frac{A_{i}E_{s}}{l_{i}}$$

The term $T_{i}$ denotes the lateral deformation term which is different in two theories. It is defined as

(4) $$T_{i}=\frac{12E_{s}I_{i}}{l_{i}^{3}}$$

and

(5) $$T_{i}=\frac{1}{\frac{l_{i}^{3}}{12E_{s}I_{i}}+\frac{l_{i}}{\kappa A_{i}G_{s}}}$$

for Euler-Bernoulli and Timoshenko beam theories, respectively (see [47] for more details). By considering these terms and implementing the superposition method, the final stiffness matrix for the 3D re-entrant structure could be obtained as $\left[K\right]=\left[\begin{array}{ccc} \left[K_{1}\right] & \left[K_{2}\right] & \left[K_{3}\right] \end{array}\right]$, where partial matrices $\left[K_{1}\right],\;\left[K_{2}\right]$ and $\left[K_{3}\right]$ are given by:

(6) $$\left[K_{1}\right]=\left[\begin{array}{cccc} 8S_{2} & 0 & -8S_{2} & 0\\ 0 & 4T_{2} & 0 & -4T_{2}\\ -8S_{2} & 0 & 4(2S_{2}+2\sin^{2}\theta S_{3}+\sin^{2}\varphi S_{5}+2\cos^{2}\theta T_{4}+\cos^{2}\varphi T_{5}) & 4C\cos\varphi \sin\varphi (-S_{5}+T_{5})\\ 0 & -4T_{2} & 4\cos\varphi \sin\varphi (-S_{5}+T_{5}) & 4(\cos^{2}\varphi S_{5}+T_{2}+2T_{3}+\sin^{2}\varphi T_{5})\\ 0 & 0 & -8(\sin^{2}\theta S_{3}+\cos^{2}\theta T_{4}) & 0\\ 0 & 0 & 0 & -8T_{3}\\ 0 & 0 & 8\cos\theta \sin\theta (-S_{3}+T_{4}) & 0\\ 0 & 0 & 0 & 0\\ 0 & 0 & 0 & 0\\ 0 & 0 & 0 & 0\\ 0 & 0 & 0 & 0\\ 0 & 0 & -4(\sin^{2}\varphi S_{5}+\cos^{2}\varphi T_{5}) & 2\sin2\varphi (S_{5}-T_{5}) \end{array}\right]$$

(7) $$\left[K_{2}\right]=\left[\begin{array}{cccc} 0 & 0 & 0 & 0\\ 0 & 0 & 0 & 0\\ -8(\sin^{2}\theta S_{3}+\cos^{2}\theta T_{4}) & 0 & 8\cos\theta \sin\theta (-S_{3}+T_{4}) & 0\\ 0 & -8T_{3} & 0 & 0\\ 16(S_{1}+\sin^{2}\theta S_{3}+\cos^{2}\theta T_{4}) & 4\sin2\theta (S_{3}-T_{4}) & 4\sin2\theta (S_{3}-T_{4}) & 0\\ 4\sin2\theta (S_{3}-T_{4}) & 8(\cos^{2}\theta S_{3}+T_{3}+\sin^{2}\theta T_{4}) & 0 & 0\\ 4\sin2\theta (S_{3}-T_{4}) & 0 & 8(\cos^{2}\theta S_{3}+T_{3}+\sin^{2}\theta T_{4}) & 0\\ 0 & 0 & 0 & 8S_{2}\\ 0 & 0 & 0 & 0\\ -8(\sin^{2}\theta S_{3}+\cos^{2}\theta T_{4}) & 8\cos\theta \sin\theta (-S_{3}+T_{4}) & 0 & -8S_{2}\\ 0 & 0 & -8T_{3} & 0\\ 0 & 0 & 0 & 0 \end{array}\right]$$

(8) $$\left[K_{3}\right]=\left[\begin{array}{cccc} 0 & 0 & 0 & 0\\ 0 & 0 & 0 & 0\\ 0 & 0 & 0 & -4(\sin^{2}\varphi S_{5}+\cos^{2}\varphi T_{5})\\ 0 & 0 & 0 & 2\sin2\varphi (S_{5}-T_{5})\\ 0 & -8(\sin^{2}\theta S_{3}+\cos^{2}\theta T_{4}) & 0 & 0\\ 0 & 8\cos\theta \sin\theta (-S_{3}+T_{4}) & 0 & 0\\ 0 & 0 & -8T_{3} & 0\\ 0 & -8S_{2} & 0 & 0\\ 4T_{2} & 0 & -4T_{2} & 0\\ 0 & 4(2S_{2}+2\sin^{2}\theta S_{3}+\sin^{2}\varphi S_{5}+2\cos^{2}\theta T_{4}+\cos^{2}\varphi T_{5}) & 4\cos\varphi \sin\varphi (-S_{5}+T_{5}) & -4(\sin^{2}\varphi S_{5}+\cos^{2}\varphi T_{5})\\ -4T_{2} & 4\cos\varphi \sin\varphi (-S_{5}+T_{5}) & 4(\cos^{2}\varphi S_{5}+T_{2}+2T_{3}+\sin^{2}\varphi T_{5}) & 2\sin2\varphi (S_{5}-T_{5})\\ 0 & -4(\sin^{2}\varphi S_{5}+\cos^{2}\varphi T_{5}) & 2\sin2\varphi (S_{5}-T_{5}) & 4(S_{4}+2\sin^{2}\theta S_{5}+2\cos^{2}\theta T_{5}) \end{array}\right]$$

The details of obtaining the elements of the stiffness matrix are provided in the Supplementary material accompanying the paper.

#### 2.1.2. Elastic Properties Relationships

All the unknown DOFs $q_{i}$ (i=1 to 12) can be obtained as functions of the external force $F_{UC}$ (which could be considered as $F_{x}$ or $F_{y}$ based on which direction we want to calculate the mechanical properties). This can be done by inverting the final stiffness matrix $\left[K\right]=\left[\begin{array}{ccc} \;\left[K_{1}\right] & \;\left[K_{2}\right] & \;\left[K_{3}\right] \end{array}\right]$ and multiplying it by the force vector. The elastic modulus of the unit cell can be calculated according to the basic definition of elastic modulus as follows:

(9) $$E_{UC}=\frac{F_{UC}L_{UC}}{A_{UC}\delta_{UC}}$$

where$\;F_{UC}$,$\;L_{UC}$,$\;A_{UC}$,$\;\delta_{UC}$, and $E_{UC}$ are the applied load, length, cross-sectional area, deformation in the load direction, and elastic modulus of the re-entrant unit cell, respectively. By considering $F_{UC}=F_{y}$,$\;\delta_{UC}=2q_{1}$, $L_{UC}=H$ and $A_{UC}=L^{2}$, the elastic modulus of the re-entrant unit cell in the y-direction can be obtained as:

(10) $$E_{y}=\frac{F_{y}.H}{2L^{2}q_{1}}$$

In addition, Poisson’s ratio of the unit cell for this loading condition can be found as:

(11) vyx=q11q1

By calculating $q_{1}$ and $q_{11}$ from the solution of the system of equations and inserting them in Equations (10) and (11), the normalized elastic modulus and Poisson’s ratio relationships of the re-entrant unit cell for Euler-Bernoulli theory are obtained as:

(12) $$\frac{E_{y,EB}}{E_{s}}=\frac{32Ht^{4}\cos\theta }{L^{2}\left(16Ht^{2}\cos\theta +L\left(L^{2}+2t^{2}-2t^{2}\cos2\theta +16t^{2}\sin\theta \right)\right)}$$

(13) $$\nu_{yx,EB}=\frac{L^{2}\left(-L^{2}+2t^{2}+2t^{2}\cos2\theta \right)\tan\theta }{H\left(16Ht^{2}\cos\theta +L\left(L^{2}+2t^{2}-2t^{2}\cos2\theta +16t^{2}\sin\theta \right)\right)}$$

where EB denotes the Euler-Bernoulli beam theory. By considering $F_{UC}=F_{x}$,$\;\delta_{UC}=2q_{11}$, $L_{UC}=L$ and $A_{UC}=HL$, the elastic modulus of the re-entrant unit cell in the x-direction can be calculated as:

(14) $$E_{y}=\frac{F_{x}}{2Hq_{11}}$$

In addition, Poisson’s ratio of the unit cell for this loading condition can be found as:

(15) vyx=q1q11

Similar to the y-direction, by substituting the results of calculated $q_{1}$ and $q_{11}$ into Equations (14) and (15), the normalized elastic modulus and Poisson’s ratio relationships of the re-entrant unit cell for Euler-Bernoulli theory could be found as:

(16) $$\frac{E_{x,EB}}{E_{s}}=-\frac{64t^{4}\cos^{3}\theta \left(L^{2}+2t^{2}-2t^{2}\cos2\theta \right)}{HL\left(-L^{4}-14L^{2}t^{2}-2t^{4}+\left(L^{4}-t^{4}\right)\cos2\theta +2t^{2}\left(-L^{2}+t^{2}\right)\cos4\theta +t^{4}\cos6\theta \right)}$$

(17) $$\nu_{xy,EB}=\frac{H\left(L^{4}-2t^{4}-4L^{2}t^{2}\cos2\theta +2t^{4}\cos4\theta \right)\sin2\theta }{L\left(-L^{4}-14L^{2}t^{2}-2t^{4}+\left(L^{4}-t^{4}\right)\cos2\theta +2t^{2}\left(-L^{2}+t^{2}\right)\cos4\theta +t^{4}\cos6\theta \right)}$$

By considering the lateral deformation term ($T_{i}$) for Timoshenko beam theory which has been defined as Equation (5) in the stiffness matrix (Equations (6)–(8)), all the relationships could be transformed into relationships based on Timoshenko beam theory. After this transformation, the elastic modulus and Poisson’s ratio relationships in the y-direction for Timoshenko beam theory can be obtained as follows:

(18) $$\frac{E_{y,T}}{E_{s}}=\frac{32H\kappa t^{4}\cos\theta }{L^{2}\left(16H\kappa t^{2}\cos\theta +L\left(\kappa L^{2}+4t^{2}+2\kappa t^{2}+4t^{2}v-2t^{2}\left(-2+\kappa -2v\right)\cos2\theta +16\kappa t^{2}\sin\theta \right)\right)}$$

(19) $$\nu_{yx,B}=\frac{L^{2}\left(\kappa \left(L^{2}-2t^{2}\right)+4t^{2}\left(1+v\right)-2t^{2}\left(\kappa -2\left(1+v\right)\right)\cos2\theta \right)\tan\theta }{H\left(16H\kappa t^{2}\cos\theta +L\left(\kappa L^{2}+4t^{2}+2\kappa t^{2}+4t^{2}v-2t^{2}\left(-2+k-2v\right)\cos2\theta +16\kappa t^{2}\sin\theta \right)\right)}$$

The shear coefficient factor of Timoshenko beam theory for square cross-section used in the relationships is

(20) $$\kappa =\frac{10\left(1+v\right)}{12+11v}$$

Similarly, the relevant equations for Timoshenko beam theory in the x-direction can be obtained as:

(21) $$\frac{E_{x,T}}{E_{s}}=\frac{\left(64\kappa t^{4}\cos^{3}\theta \left(\kappa \left(L^{2}+2t^{2}\right)+4t^{2}\left(1+v\right)-2t^{2}\left(\kappa -2\left(1+v\right)\right)\cos2\theta \right)\right)}{A_{1}}$$

where:

(22) $$\begin{array}{c} A_{1}=\{HL(\kappa^{2}L^{4}+4\kappa L^{2}t^{2}+14\kappa^{2}L^{2}t^{2}+8t^{4}+56\kappa t^{4}+2\kappa^{2}t^{4}+4\kappa L^{2}t^{2}v+16t^{4}v+56\kappa t^{4}v+8t^{4}v^{2}\\ +\left(\kappa^{2}\left(-L^{4}+t^{4}\right)+60\kappa t^{4}\left(1+v\right)+4t^{4}{\left(1+v\right)}^{2}\right)\cos2\theta +2t^{2}\left(k-2\left(1+v\right)\right)(\kappa \left(L^{2}-t^{2}\right)\\ +2t^{2}(1+v))\cos4\theta -\left(4t^{4}-4\kappa t^{4}+\kappa^{2}t^{4}+8t^{4}v-4\kappa t^{4}v+4t^{4}v^{2}\right)\cos6\theta )\} \end{array}$$

and

(23) $$\nu_{xy,T}=-\frac{A_{2}\left(\kappa L^{2}+4t^{2}\left(1+v\right)\right)\cos2\theta +2t^{4}{\left(-2+\kappa -2v\right)}^{2}\cos4\theta )\sin2\theta )}{A_{3}}$$

where:

(24) $$A_{2}=\left\{H(\kappa^{2}L^{4}+8\kappa L^{2}t^{2}+24t^{4}-8\kappa t^{4}-2\kappa^{2}t^{4}+8\kappa L^{2}t^{2}v+48t^{4}v-8\kappa t^{4}v+24t^{4}v^{2}-4t^{2}(\kappa -2\left(1+v\right)\right\}$$

(25) $$\begin{array}{c} A_{3}=L(\kappa^{2}L^{4}+4\kappa L^{2}t^{2}+14\kappa^{2}L^{2}t^{2}+8t^{4}+56\kappa t^{4}+2\kappa^{2}t^{4}+4\kappa L^{2}t^{2}v+16t^{4}v+56\kappa t^{4}v+8t^{4}v^{2}+\\ \left(\kappa^{2}\left(-L^{4}+t^{4}\right)+60\kappa t^{4}\left(1+v\right)+4t^{4}{\left(1+v\right)}^{2}\right)\cos2\theta +2t^{2}\left(\kappa -2\left(1+v\right)\right)(\kappa \left(L^{2}-t^{2}\right)+2t^{2}(1+v))\cos4\theta -\\ \left(4t^{4}-4\kappa t^{4}+\kappa^{2}t^{4}+8t^{4}v-4\kappa t^{4}v+4t^{4}v^{2}\right)\cos6\theta )\}. \end{array}$$

It is worth noting that all the final relationships have been presented for the specific condition of θ=φ, otherwise, the relationships became very lengthy.

### 2.2. Numerical Analysis

In the second stage of this study, the focus is on the biomedical application of the analyzed 3D re-entrant structure to improve the micromotion problems in implants, particularly in femoral hip joint implants. In this subsection, the detailed steps of designing and FE analysis of femoral implant constructed from 3D re-entrant lattice structures are described. Three types of porous implants with positive, negative and graded Poisson’s ratios are constructed for this aim.

#### 2.2.1. Methodology of the Implant Design

In this study, the upper half of human femur bone with a total length of 25.83 cm has been considered for numerical modelling and analysis (Figure 3a). The femur bone geometry was obtained from computed topography (CT) scans of a 40-year-old healthy male. Using MATLAB (MathWorks, Natick, MA, USA), a 3D model consisting of external surfaces of the femur bone geometry was constructed. The obtained surfaces were then transferred to SolidWorks (Dassault Systèmes, Vélizy-Villacoublay, France), and the final model was polished there to provide a smooth and easy-to-use femur bone computer-aided design (CAD) model.

The main parameters used for implant design were chosen based on the values suggested in the literature. The head diameter was set to 40 mm which is in the ranges of 22-45 mm as suggested by Charnley et al. [48] and McKnee et al. [49]. Moreover, the length and diameter of the neck were 18 mm and 13 mm, respectively, which are in the ranges suggested in the literature: 10 mm (short-neck) to 40 mm (long-neck) for the length of the neck and 13–30 mm for the diameter of the neck [50,51]. The length of the intramedullary stem was set to 125 mm which lies in the advised range of 120–180 mm [52]. Moreover, the neck-shaft angle was 140° which is in the range of 135–145° performed in previous studies [52]. 

**Figure 4:**
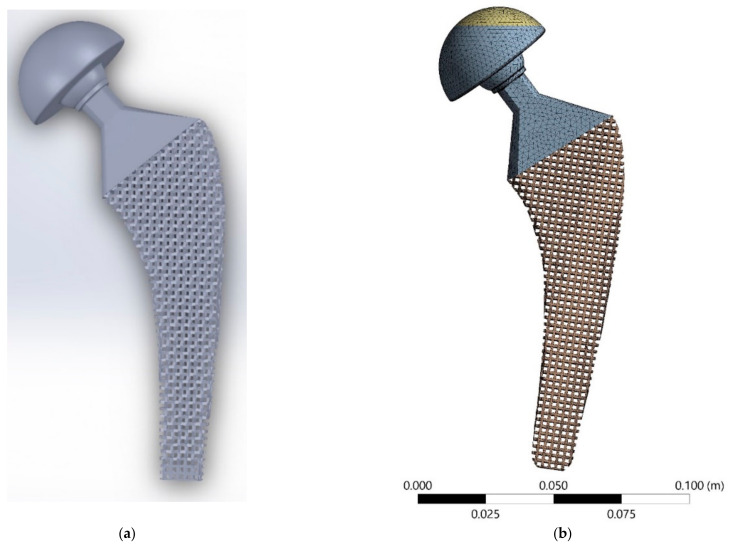

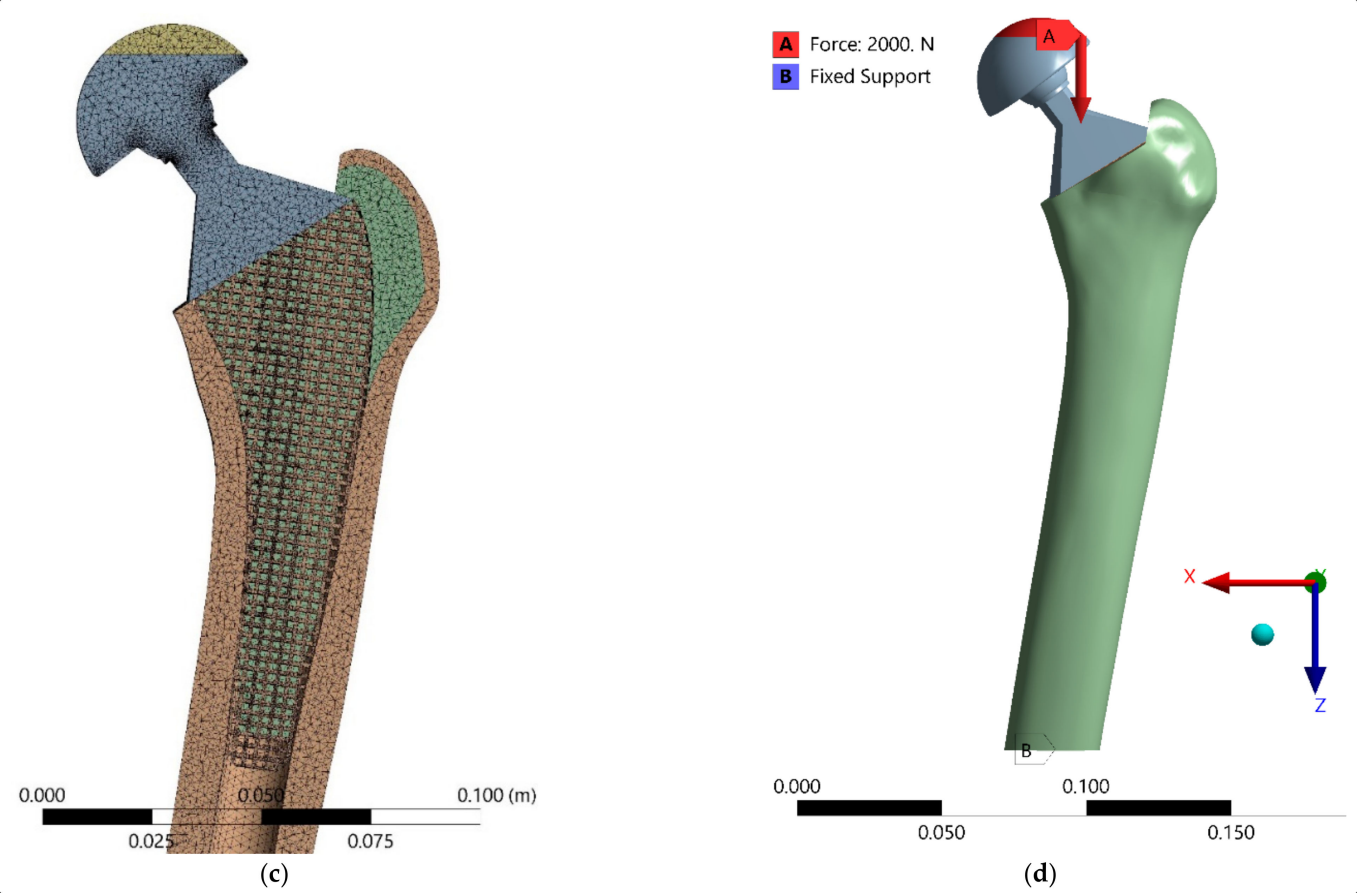
(**a**) CAD model and (**b**) meshed finite element (FE) model of meta-implant based on 3D re-entrant structure. (**c**) Final assembly of the implant and femur bone model with its corresponding mesh (**d**) Boundary condition and applied load on the implant and femur bone model.

For comparison purposes, first, a solid implant is modelled. It is worth noting that to have more precise numerical analysis, both the cortical and trabecular parts of the femur bone have been considered in the FE modelling. To create a compatible geometry for the femoral implant, the anatomy of the femur bone has been considered as the basic geometry of the implant. For this aim, the implant external edges and surface (Figure 3b) have been designed in compliance with the internal faces of the cortical part of the femoral bone model (Figure 3a). The total length of the modelled implant was 16.72 cm. Implementing this concept minimizes the potential gap occurrence at the femur bone and implant interface when the bone/implant assembly undergoes external loading. The final assembled model consisting of cortical bone, trabecular bone, and the solid implant is shown in Figure 4c. The material properties used for the solid implant was $E_{s}=113.8$ GPa and $v_{s}=0.342$ which is based on Ti-6Al-4V as well-known implant material alloy. Mesh sensitivity analysis was performed for six different element sizes (1 mm, 0.7 mm, 0.4 mm, 0.3 mm, 0.2 mm, and 0.1 mm). Based on the results, the element sizes in the range of 0.3–0.7 mm that was used in this study gave sufficiently accurate results. In fact, the chosen element size range gave less than 1.4–4% (depending on the case) difference in results as compared to the smallest element size of 0.1 mm.

#### 2.2.2. Meta-Implant Design

For the case of meta-implants, we need to connect the mechanical properties of the human femur bone and our analytical solution of the re-entrant structure to provide a lattice structure for the hip implant (stiffness of which accommodates to that of human bone). The main mechanical parameters required to create a meta-implant that is compliant with its surrounding femur bone of study are elastic modulus and Poisson’s ratio. The implant elastic modulus controls the stress flow and its distribution in the implant. While elastic modulus can affect the micromotion of the implant/bone interface in the longitudinal direction, Poisson’s ratio can affect the micromotion in the transverse direction. A lot of works in the literature have studied the mechanical properties of the human femur bone [53,54,55,56,57]. In this study, the values of 16.7 GPa and 0.155 GPa have been considered for the elastic modulus of respectively the cortical and trabecular bones based on a similar study [58]. In addition, the values of respectively 0.3 and 0.25 have been considered for the Poisson’s ratio of cortical and trabecular bones [59]. 

According to our analytical solution, the re-entrant structure provides much larger elastic modulus ranges in the x-direction as compared to the y-direction (see Results Section). Therefore, to gain the elastic modulus of 16.7 GPa for the meta-implant, the analytical relationships for the elastic modulus of the unit cell in the x-direction (Equation (21)) has been used. Therefore, the x-direction of the re-entrant structure was aligned with the human femur bone shaft central axis at the lowest level of the hip implant. The low number of unit cells at the bottom part of the hip implant is the reason for this alignment as it provides better contact between unit cell struts and the bone internal surfaces. By considering the elastic modulus value of $E_{s}=113.8$ GPa for Ti-6Al-4V as a well-known implant material alloy, which has been used for normalizing the analytical relationships, the suitable normalized elastic modulus of the re-entrant structure is obtained as 0.1467. Since the re-entrant structure provides almost the same values of elastic modulus for positive or negative values of θ and φ (see Results Section), two different hip meta-implants with positive and negative Poisson’s ratios are constructed to have a deeper understanding of the effect of Poisson’s ratio on the micromotion at the bone-implant interface.

#### 2.2.3. Graded Meta-Implant

Based on the previous studies [60,61] on the femur implant and the results of this study for solid and femoral meta-implant (with either completely positive or negative unit cells) models, it is clear that the stress distribution in the hip implant varies from negative values to positive values due to the tensile and compressive load distribution in the implant (see Section 3). The main problem in the meta-implants with either completely positive or negative unit cells is that the parts of the implant with positive Poisson’s ratio which have tensile stress distribution tend to separate from the bone surface at the contact surfaces. A behaviour also occurs when the parts of the implant with negative Poisson’s ratio are subjected to compressive stress. This problem can be resolved by implementing negative Poisson’s ratio unit cell of re-entrant structure in the areas of the implant which have tensile stress distribution on one hand and using negative Poisson’s ratio unit cell of re-entrant structure in the areas of the implant which have compressive stress distribution on the other hand. To apply this approach to our meta-implant, a gradient structure which varies from positive Poisson’s ratio at one side of the implant to negative Poisson’s ratio at the other side of the implant is designed and constructed. The variation of Poisson’s ratio in the re-entrant structure was achieved by changing the values of angle θ only (θ=−8.5° for Poisson’s ratio of 0.3 and θ=8.5° for Poisson’s ratio of −0.3). This allowed for a smoother distribution of mechanical properties in the final implant structure. 26 rows of the re-entrant unit cell with dimensions of 5 × 5 × 5 mm^3^ have been used for generating the graded meta-implant.

#### 2.2.4. FE Analysis

The FE analyses of the implants under static compressive loading condition were performed using the ANSYS Workbench (ANSYS, Canonsburg, PA, USA) software (Version 19.3). To better simulate the real human femur loading condition, the femur bones were angled at 12° flexion and 12° adduction, and the lower part of the femur bone (middle shaft) was fixed in all the directions. The femoral head was loaded by applying a 2000 N compressive load to the head of the femur implant. The loading and boundary conditions considered in this study are based on the basic experimental setups which have been used in several experimental studies of human femur hip implants [58,62,63].

The contact condition between the cortical and trabecular bones was considered as a bonded constraint. On the other hand, to track the micromotion between the implant external surfaces and bone internal surfaces, the contact condition between the bone and the implant was considered as frictional surface-to-surface contact allowing contact, sliding, and tensile separation to occur. The friction coefficient of 0.3 was applied for the frictional contact conditions [64]. The 3D solid elements were used for discretization of the model in the ANSYS Workbench software, and fine mesh size with the smooth transition was used for providing better mesh quality especially at the edge of the implants. The FE model of the meta-implant and the assembled model with femur bone are presented in Figure 4. The total number of elements for solid implant assembly was 463,634. Since the lattice structures in the meta-implants consist of a large number of thin struts (t = 1.04 mm), the mesh size for meta-implants was reduced to about 1/4 of that in the solid implant. As a result, the total number of elements for meta-implants with positive and negative Poisson’s ratio were 1,393,745 and 1,563,915, respectively. For the case of graded meta-implants, due to its geometrical complexity and computational costs, the mesh size needed to be increased, and the final total elements number reached 866,600 elements.

## 3. Results

### 3.1. Effect of Relative Density on the Unit Cell Mechanical Properties

**Figure 5:**
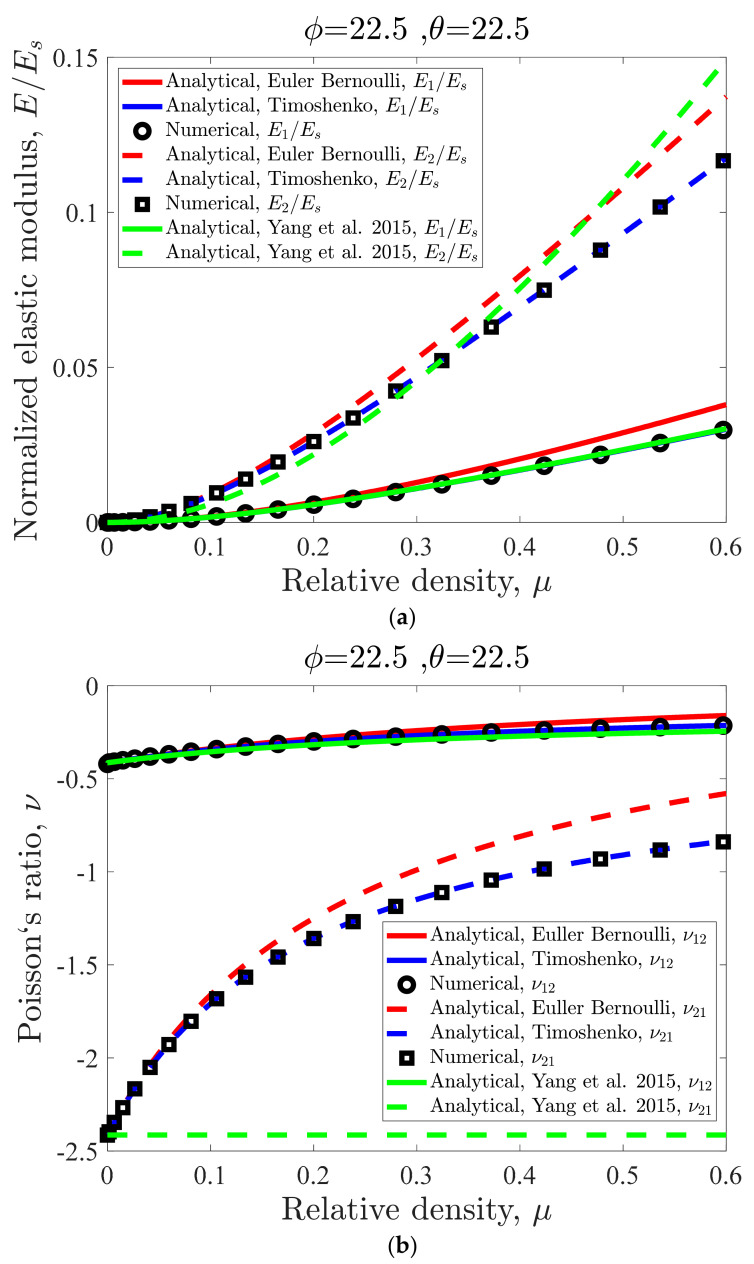
Mechanical properties: (**a**) normalized elastic modulus, (**b**) Poisson’s ratio.

Variations in normalized elastic modulus $E/E_{s}$ for the special case of θ=φ=22.5° is demonstrated in Figure 5. The results show that in all ranges of relative density, the elastic modulus is almost quadruple in the x-direction when compared to that in the y-direction (Figure 5a). For instance, at a relative density of 0.6, relative elastic modulus based on Timoshenko beam theory in the x-direction is 0.117, while it is 0.03 in the y-direction. The elastic modulus curve based on Timoshenko beam theory is always lower than the curve obtained based on Euler-Bernoulli beam theory (Figure 5a). Their difference, however, does not exceed 14.76%. In the y-direction, the curve obtained by Yang et al. [38] theory overlaps with the Timoshenko results obtained here. However, in the x-direction, the curve obtained by Yang et al. [38] overlaps neither with the Timoshenko analytical results nor with the Euler-Bernoulli analytical results obtained in this paper (Figure 5a). Nevertheless, in high relative densities (i.e., μ>0.3), the results of Yang et al. theory in the y-direction are close to the curves obtained in this paper. It is important to note that the FE results always overlap with the analytical results based on Timoshenko beam theory (Figure 5a). 

As for the Poisson’s ratio, the results show that even though $\nu_{yx}$ is always negative, relative density has very little effect on its value (Figure 5b). In fact, $\nu_{yx}$ only changes in the range of −0.2143 and −0.4132. However, changing relative density from 0.6 to almost zero changes $\nu_{xy}$ from −0.8393 to −2.414. Timoshenko beam theory usually predicts greater negative Poisson’s ratio (in an absolute sense) when compared to the Euler-Bernoulli beam theory. Regardless of direction, the curves obtained by Yang et al. [38] do not have any overlap with neither the analytical Timoshenko results nor with the analytical Euler-Bernoulli results. A more prominent difference between our results and those of Yang et al. [38] is for $\nu_{xy}$. While Yang et al. [38]’s theory gives the constant value of −2.414 for $\nu_{xy}$ in all the relative densities, the analytical results in the current work show that $\nu_{xy}$ changes from −0.8393 to −2.414 (Figure 5b). Similar to elastic modulus results, the FE results always overlap with the analytical results based on Timoshenko beam theory.

### 3.2. Effect of Internal Angle on the Unit Cell Mechanical Properties

**Figure 6:**
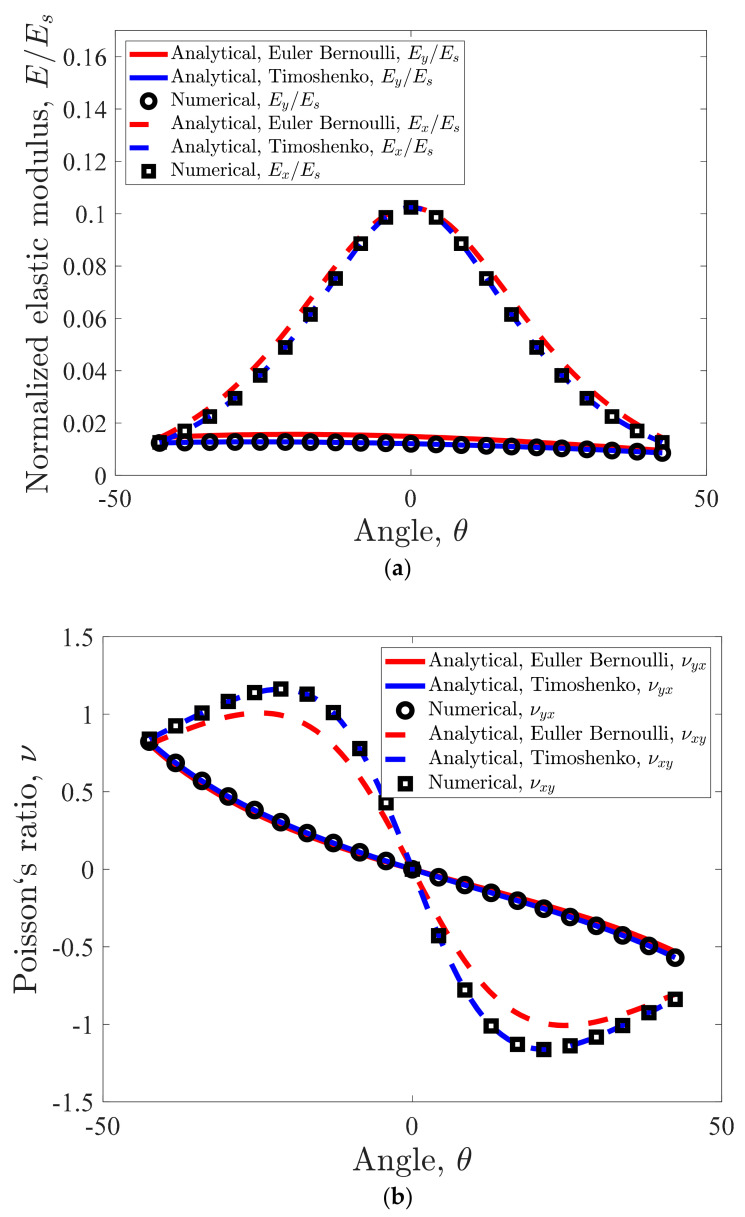
Effect of angle on mechanical properties for r = 0.8 mm, H = L = 5 mm: (**a**) normalized elastic modulus, (**b**) Poisson’s ratio.

The internal angles θ and φ are other important factors in geometrical characteristics of the unit cell. Due to the fact that the re-entrant structure is homogenized by considering $l_{1}=l_{2}$ and $h_{1}=h_{2}$, in conjunction with the similarity of the relationships for these conditions, the angles θ and φ are set equal to each other here as well as throughout the study. The ratio of struts thickness to the height (or length) of the unit cell for which the mechanical properties graphs are plotted (Figure 6) is r/*H* = 0.16. As for the elastic modulus, the internal angle has an almost negligible effect in the y-direction. However, in the x-direction, the relative elastic modulus in θ=0° is almost 8-fold of that in θ=42.5° (0.1024 as compared to 0.0126). It must be noted that the elastic modulus curves are semi-symmetrical with respect to θ=0° (Figure 6a).

As for the Poisson’s ratio, the internal angle has a significant effect on both $\nu_{xy}$ and $\nu_{yx}$ (Figure 6b). $\nu_{xy}$ is completely symmetrical and $\nu_{yx}$ is almost symmetrical with respect to the point $\left(\theta ,\;\nu \right)=\left(0,\;0\right)$. The little asymmetrically observed for $\nu_{yx}$ is caused by the differences in the lengths $h_{1}$ and $h_{2}$ for positive and negative extents of φ and θ which has effects on mechanical properties. While from θ=−42.5° to θ=42.5°, $\nu_{yx}$ changes steadily (almost linearly) from 0.8227 to −0.5701, $\nu_{xy}$ has more extreme variations in the angle range (Figure 6b). $\nu_{xy}$ changes from 1.162 to −1.162 in a smaller angle range of θ=−21.25° to θ=21.25°.

### 3.3. Stress and Strain Distributions

**Figure 7:**
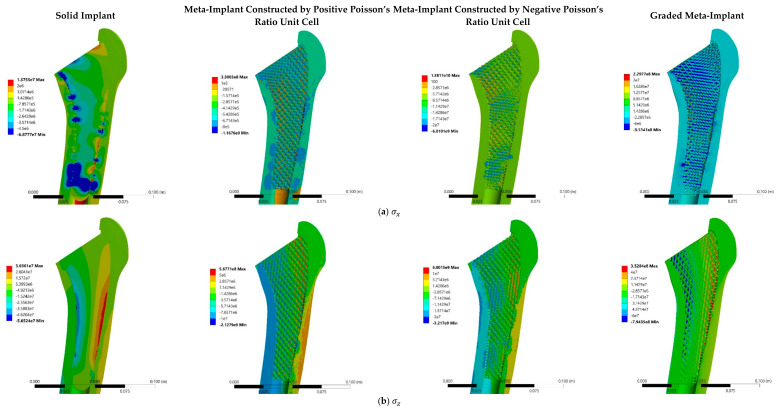

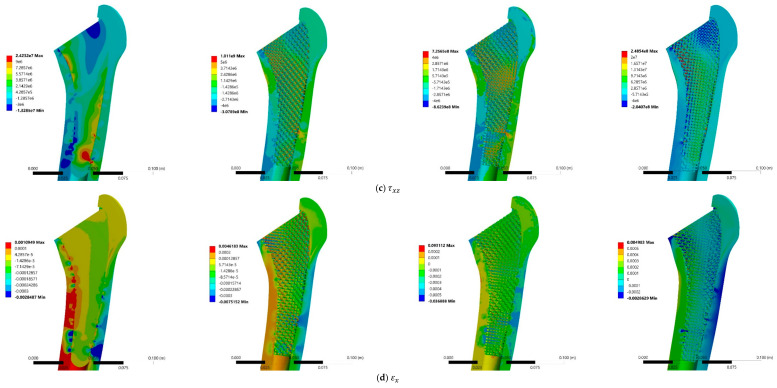

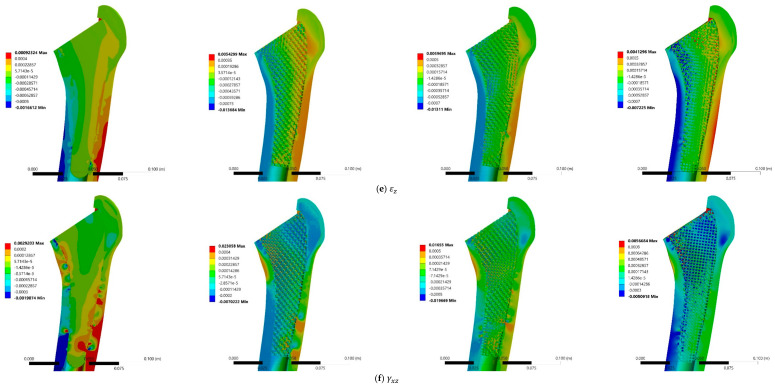
Stress (Pa) and strain distribution: (**a**) normal stress in the x direction, (**b**) normal stress in the z direction, (**c**) shear stress in the xz direction, (**d**) normal strain in the x direction, (**e**) normal strain in the z direction, and (**f**) shear strain in the xz direction. The stress and strain components are presented for the x and z directions only. This is due to the fact that due to low thickness of the implant (and a low number of unit cells) in the y-direction as well as plane-stress loading condition in the x-y plane, the stress and strains in the y-direction were negligible.

The stress and strain distributions in femur bone filled by the solid implant, the meta-implant constructed by positive Poisson’s ratio unit cells, the meta-implant constructed by negative Poisson’s ratio unit cells, and the meta-implant with the graded distribution of unit cells are shown in Figure 7. In the case of the solid implant, there is a high mismatch of $\sigma_{z}$, $\varepsilon_{x}$, and $\gamma_{xz}$ between the solid implant and the surrounding bone. In the case of the meta-implant constructed by positive unit cells, the mismatch of the noted stress ($\sigma_{z}$, $\varepsilon_{x}$, and $\gamma_{xz}$) between the implant and the bone is mitigated. Unlike the solid implant, the *local* stress distribution in meta-implant does not change uniformly. In fact, different regions of each unit cell have different stresses. Some parts of each unit cell experience stress levels similar to that of the bone, while some other parts of it experience different stress levels. From a mechanical point of view, this was well expected as some struts mainly undergo flexural loading, while some other struts mainly undergo axial loading.

In the case of structure constructed by negative unit cells, there are two improvements when compared to the structure constructed by positive unit cells. First, the stress and strain distributions in each unit cell become much more uniform locally. In other words, the stress or strain level throughout each unit cell remains almost constant. Secondly, and more importantly, the stress and strain levels in the case of structure constructed by negative unit cells is very close to that of the bone surrounding it at each location.

As for the case of the graded meta-implant, even though the stress and strain distributions (both locally inside each unit cell and as compared to bone) in the graded meta-implant are much better than the structure constructed by positive unit cells, it performs worse than the structure constructed by negative unit cells.

In addition to stress and strain distributions, the level of stress dominant in the implant is also important. Very high-stress levels can lead to early implant failure. As for the case of the solid implant, the maximum stress in the implant is $\left|\sigma_{max}\right|=68$ MPa (Figure 7a) which is relatively low, as was expected. In the case of meta-implant constructed by positive unit cells, the maximum stress level is $\left|\sigma_{max}\right|=2127$ MPa. In the case of the meta-implant constructed by negative unit cells, the maximum stress level is $\left|\sigma_{max}\right|=13,817$ MPa. In the case of the graded meta-implant, the maximum stress level is $\left|\sigma_{max}\right|=794$ MPa. This shows that the graded meta-implant experiences much lower stress levels as compared to the two other meta-implants, and that the stress the graded meta-implant experiences is in the range bearable by titanium alloys.

### 3.4. Micromotion

**Figure 8:**
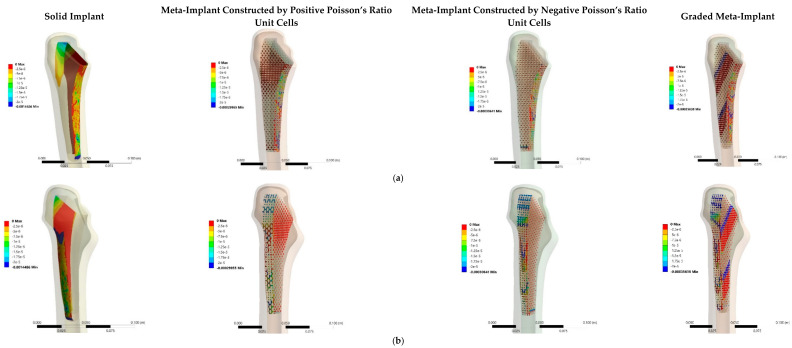

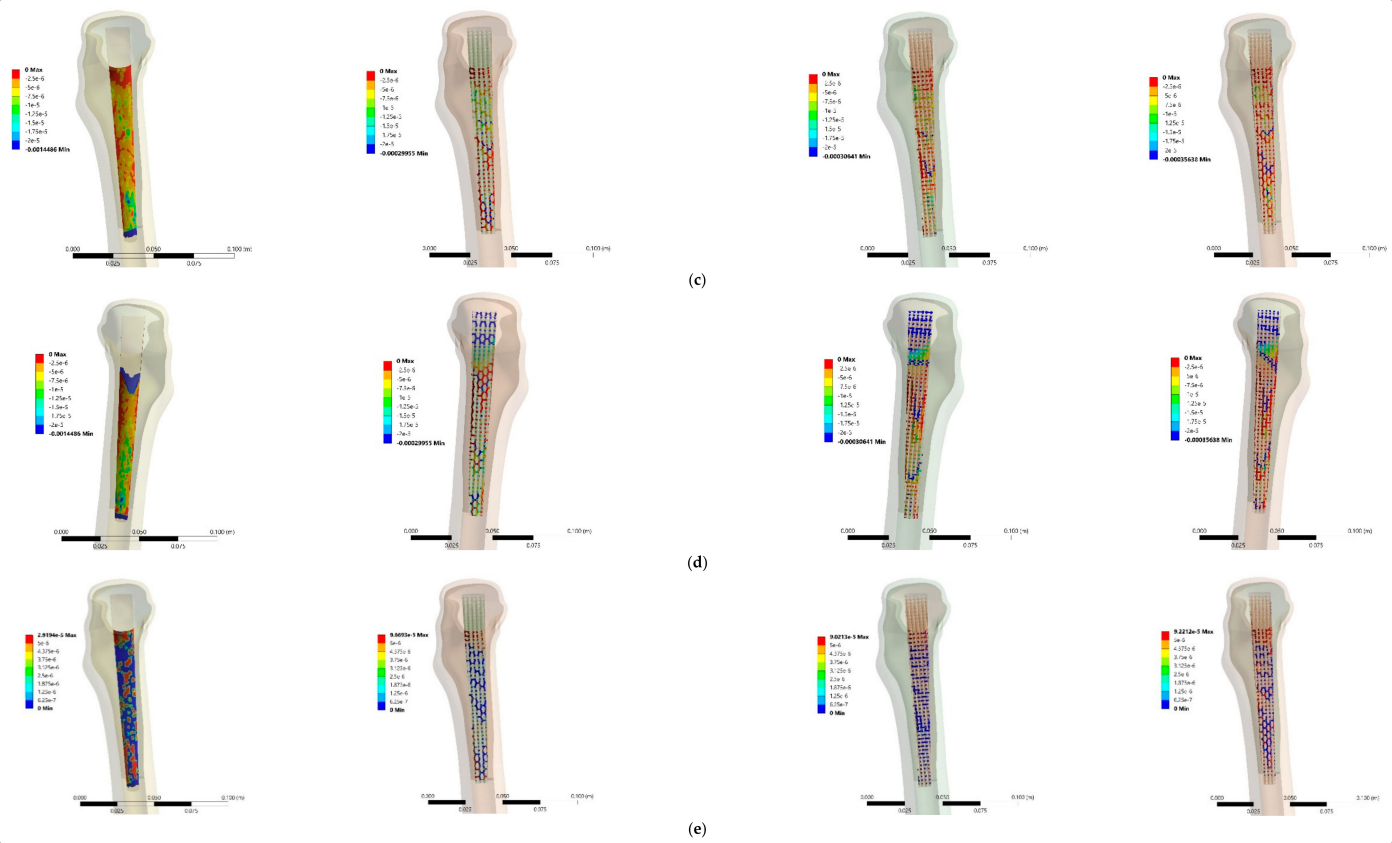

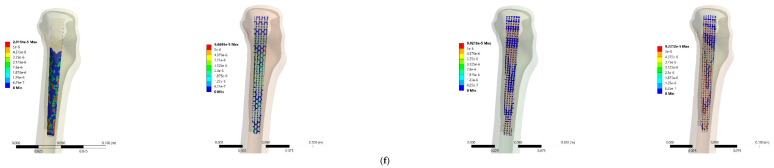
Micromotions in implant faces: (**a**,**c**) front-side gap, (**b**,**d**) back-side gap, (**e**) front-side sliding, and (**f**) back-side sliding.

**Figure 9:**
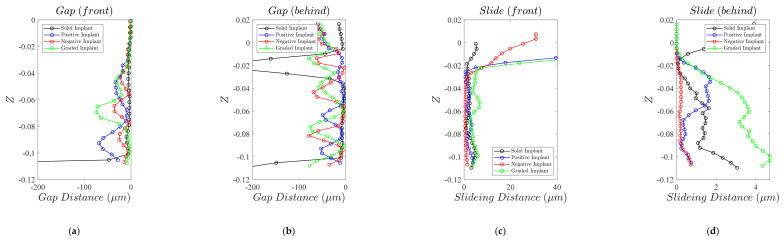
Micromotions in implant faces: (**a**) front-side gap, (**b**) back-side gap, (**c**) front-side sliding, and (**d**) back-side sliding.

Micromotion, another important factor in the durability of an implant, is compared between the four types of the implant in Figure 8. To get a more quantitative impression of comparison of micromotion between the implant and the bone, the average sliding and gap distances between the implant and the bone at each height of the implant is plotted in Figure 9. In general, except at the very top part of the meta-implant (Z>−0.02), the maximum sliding in none of the meta-implants exceed 4.5 µm. Therefore, the main micromotion comparison between the implants should be made based on the *gap* between the implant and the bone.

Except for one point, the solid implant demonstrates a near-zero gap in both back and front (the parts in compression and tension) of the implant (Figure 9a,b). Among the meta-implants, the meta-implant constructed by negative unit cells has the lowest level of the gap in the front side. The average gap between the meta-implant constructed by positive unit cells and the bone is 25.02 µm in the front side and 26.27 µm in the back side. The average gap between the meta-implant constructed by negative unit cells and the bone is 11.36 µm in the front side and 35.43 µm in the back side. Finally, the average gap between the graded meta-implant and the bone is 19.78 µm in the front side and 47.65 µm in the back side. Therefore, based on the above-mentioned results, the meta-implants constructed by positive Poisson’s ratio and the meta-implants constructed by negative Poisson’s ratio provide the minimum and maximum average values of the gap at the front side of the implants, respectively. On the other hand, the meta-implant based on the positive Poisson’s ratio and graded structures present the minimum and maximum average values of the gap at the back side of the implants, respectively. 

Based on the results of the gap and sliding distributions presented in Figure 8 and Figure 9, the extent of sliding distance (≤5 μm) at both sides of implants is negligible when compared to the gap distance extent (≤75 μm ) in most parts of the implant. Therefore, the micromotion in implant, which is defined as the vector resultant of sliding and gap distances, could be well approximated by only the gap distance of the implant at the contact surface. The distribution of gap distance in the solid implant is mostly uniform and varies between 0 and 15 μm at different heights of the implant at both sides (Figure 9a,b). As shown in Figure 9b, a huge gap of ≥200 μm occurs between the solid implant and the bone due to discontinuous contact surface at the back side of the solid implant at the height of z≅−0.02 m. In comparison with the meta-implant constructed by positive Poisson’s ratio unit cells, the distribution of gap distance in the graded meta-implant and the meta-implant constructed by negative unit cells is more uniform (and with the sinusoidal shape). At the back side of the implant, the gap value of the graded and negative Poisson’s ratio meta-implant varies between 0 and 75 μm sinusoidally with the height of the implant. In the front side, this oscillating behaviour could be only observed for the meta-implant with negative Poisson’s ratio which varies between 0 and 25 μm.

## 4. Discussions

### 4.1. Analytical Model

Considering the behaviour of the re-entrant unit cell as the sum of the behaviour of individual DOFs for an analytical solution, which works as one of the most accurate approaches used for obtaining the analytical relationships for strut-based lattice structures and metamaterials. This technique, by considering all possible deformations of the struts at the joints in the lattice structure, provides comprehensive and accurate analytical solutions for the mechanical properties of lattice structures. The results of this study also confirmed that the obtained relationships for normalized elastic modulus and Poisson’s ratio of re-entrant structure based on this technique present satisfactory outcomes in comparison to other approaches presented in the literature [65]. Moreover, the general analytical solution based on this approach gives this opportunity to implement and compare the results of both the Euler-Bernoulli and Timoshenko beam theories simply by changing the corresponding parameters ($S_{i}$ and $T_{i}$). As shown in Figure 5, the Timoshenko beam theory gives much more accurate results in comparison with the Euler-Bernoulli beam theory due to the fact that it takes the shear deformation effect in the struts into account. Therefore, the Timoshenko beam theory allows for more flexibility in the structure. Hence, as compared to Euler-Bernoulli beam theory, Timoshenko beam theory predicts lower and higher values for the elastic modulus and Poisson’s ratio, respectively. Based on the aforementioned facts, we observed significant improvement in the final analytical solution in comparison with previous works [35,38], especially for normalized elastic modulus in the x-direction and $v_{xy}$. According to the final relationships, $v_{xy}$ is dependent on the relative density and angles φ and θ. However, the $v_{xy}$ relationship presented by Yang et al. [38] is independent of relative density or strut thickness which is far from accurate especially for high values of relative density.

### 4.2. Implant Macro-Geometry

Hip implant geometry is one of the most important parameters which can affect the stress-strain and micromotion distribution at the bone-implant interface. There are lots of studies in the literature which have focused on the design and optimization of the hip implant geometry to improve the fixation and to reduce the micromotion at the implant surface [39,40]. Using the internal geometry of human femur bone for designing the shape of the implant could be one of the suitable methods to have better contact between the implant and bone surfaces. The superiority of implants with bone-compliant geometry has been shown in previous studies such as [66,67]. As mentioned in Results Section (Section 3.4) and the results demonstrated in Figure 9, it is clear that the gap between the bone and implant at their interface for the solid implant is significantly low which can be attributed to good match of the geometry of implant’s external surface and femur bone’s internal surface. Due to its high stiffness as well as extremely low micromotion extent both of which create undesirable conditions for bone growth, the solid implant is not a good choice for hip implants.

### 4.3. Stress and Strain Distributions

By considering Ti-6Al-4V alloy for implants material, a large difference occurs between the value of elastic modulus of the femur bone and solid implant. This fact causes nonhomogeneous stress and strain distributions in the implant, especially at the implant-bone contact regions. For implantation with the cement-less condition (press-fit), sliding and gap distances occurring at the contact surface of the bone and solid implant during loading lead to local stress and strains at the implanted area (Figure 7) which can cause damage to the bone internal surface. Implementing lattice structures gives the opportunity of reducing the difference of elastic modulus between the bone and implant. It is worth noting that the porous structure at the contact surface of the implants and bone also contributes to improvement in bone ingrowth. That is why in early scientific studies as well as in many of today’s industrial implants, solid implants with the porous surface are used [68,69,70]. As shown in Figure 7b,c, lattice structures with adjusted elastic modulus to the bone elastic modulus improve the smoothness of stress-strain distribution in the implant especially at the interface of the bone and implant. Although the sliding between the bone and implant due to press-fit condition could still create some local stress and strain mismatch, a significant enhancement in the uniformity of stress and strain was observed in the meta-implant in comparison to the solid implant. The main reason for this fact could be the relaxation of meta-implant surfaces which occurs due to the local deformations of lattice structures struts at their contact regions with the bone. These deformations of strut beams relieve the local strain and stress which is one of the major problems observed in the solid implant.

### 4.4. Micromotion

Although decreasing micromotions at the implant-bone interface can have positive influences on the bone ingrowth, eliminating the micromotion from the bone and implant interface altogether can have negative effects on bone ingrowth and fixation durability. Burke et al. [71] have demonstrated that the micromotions of 40 µm allow the formation of woven bone within a porous titanium wire surface. Therefore, complete elimination of gap and sliding distances (the main parameters of micromotion) at the implant surface could have negative effects on bone ingrowth at the bone-implant interface. This also means that the solid implant, due to its very small values of micromotion, can raise some problems for bone ingrowth in comparison with meta-implants which provide sufficient values of micromotion at contact surfaces. On the other hand, the uniform oscillation of the gap value between 0 and 75 μm at the back side of the graded meta-implant and the meta-implant constructed by negative unit cells could satisfy the required micromotion extent for bone ingrowth at the bone-implant contact surface. The variation of the gap at the front side of the implant constructed by negative Poisson’s ratio unit cell is in the range of 0 to 30 μm, while this range for the graded meta-implant is 0 to 85 μm which could meet the suitable average micromotion requirement for successful bone ingrowth. In addition, the large internal pores, as well as struts deformations of all the meta-implants, could mitigate the micromotion at critical points of the implant such as at z≅−0.02 m in which the solid implant demonstrated huge micromotion values due to the sudden change in the geometry of the implant surface. Since most implants available in the market for hip implant replacement are manufactured based on predesigned geometries (not anatomical geometries), this fact could be considered as a strong advantageous point for meta-implants which are capable of mitigating micromotion at regions in which the implant external surface and the cortical bone internal surface are not well compatible.

### 4.5. What Micro-Structure to Choose?

Thanks to the anatomical geometry of implants and the adjusted elastic modulus of the meta-implants, all of the meta-implants based on the introduced unit cell gave acceptable stress and strain levels in the implantation area. Nevertheless, the meta-implant based on graded unit cell distribution presented smoother stress-strain distribution in comparison to the other meta-implants. Moreover, the graded meta-implant gave minimum areas of local stress and local strain concentration at the contact region of the implants with the bone internal surfaces. Among all the cases, the graded meta-implant also gave micromotion levels which were the closest to value reported to be desirable for bone growth (40 µm) [71]. The maximum level of stress in the graded meta-implant was also the lowest among all the meta-implants and within the range that is acceptable for most titanium alloys. 

### 4.6. Further Improvements and Limitations

One of the most important parameters which affect the accuracy of analytical results is the effect of considering the effective lengths of struts in a unit cell. In higher values of relative densities, the struts get thick, and the overlapping of the struts at the joints should be taken into account in the analytical solution. By defining the effective length of the struts according to their orientation and thickness, the analytical solution could predict more accurate results for the actual condition of the lattice structure [72]. Another noteworthy parameter that can enhance the results is calculating the exact relationship for the relative density of the unit cell. The exact relationship of relative density by considering the multiple counting effect of struts gives this opportunity to calculate the exact value of the strut thickness for specific mechanical properties of lattice structure [73]. As another note, the re-entrant unit cell studied in the work, as it shares struts with its neighbouring unit cells, is not an ideal structure for creating a graded structure such as the graded meta-implant designed in this study. Thus, the change of angle in the graded structure could cause problems in connecting the shared struts of adjacent unit cells. Hence using a structure such as idealized re-entrant structure [72] could solve the problems and enhance the results for the graded meta-implant. 

It must be also noted that there must be a balance between the micromotion, stress, and stiffness as the bone growth is driven by the mechanical load, which means that the elastic force acting on the bone must be somehow maintained. The effect of implant-bone interaction is usually termed as the primary stability of the implant and is closely related to the stiffness. Moreover, not only the hip stem is important, but more often, the acetabular component fails due to aseptic loosening. For real-life design, such limitations and considerations should also be taken into account, see [74,75] for more information. Furthermore, it must be mentioned that the analytical approach cannot capture local changes in structure, large displacements due to local interactions, and local buckling. However, such effects are important for the proper design of meta-implant. To address such issues, explicit FE modelling [76], as well as multi-scale numerical modelling [20,77], are very beneficial. 

As mentioned before, the loading and boundary conditions considered in this study for hip bone are based on the loading and boundary conditions of commonly used experimental setups in several experimental studies of femur hip implants [58,62,63]. The simplicity and practical condition of this setup create an opportunity for future experimental studies on this design. In addition, there are some techniques available for evaluating the strain and micromotion at the bone-implant interface such as linear variable displacement transducer (LVDT) sensors, μ-CT (micro-CT) imaging, and radiopaque markers [5,78]. These techniques allow for measuring the stem and bone deformation with acceptable accuracy. In particular, the micro-CT (μ-CT) imaging technique could be very beneficial for measuring the micromotion in the latticed regions due to its high resolution which makes it possible to measure micromotion in a wide area and with good precision [79]. As the final note, it must be mentioned that to verify the effectiveness of the proposed designs, implementation and evaluation of AM patient-specific hip meta-implants on several patients is important. Depending on the patient’s specific requirements, the range of Poisson’s ratio in each unit cell of the gradient meta-implant, as well as the thickness of struts, can be changed. A numerical package can be developed to address the patient individuality and, consequently, the non-identical design of the meta-implants.

## 5. Conclusions

A hip-joint meta-implant with an appropriate lattice structure can improve the stress and strain distributions in the implant and surrounding bone tissues. Moreover, designing an accurate and precise graded lattice structure for the implant can enhance implant deformation as well as provide suitable micromotion for bone ingrowth at the bone-implant interface. This study focused on designing a graded lattice hip meta-implant by implementing a well-known 3D re-entrant auxetic metamaterial to enhance the micromotions at the bone-implant contact surfaces. To reach this aim, analytical relationships for mechanical properties of the 3D re-entrant structure were obtained using a more accurate analytical approach when compared to methods previously used in the literature. The results demonstrated a huge improvement in the analytical solution which overlapped with FE results. One solid implant and three meta-implants were designed based on the anatomy of the human femur bone. The new exact relationships for mechanical properties of 3D re-entrant unit cells were implemented to create three meta-implants with positive, negative, and graded Poisson’s ratios. The numerical results of the implants under compressive loading conditions showed that the meta-implants could provide enhanced stress and strain distributions in the implant and its contact regions with the bone internal surface. Although the micromotion values for all the meta-implants were within appropriate ranges for bone ingrowth, the meta-implants with negative and graded Poisson’s ratios gave a more compatible and uniform distribution of micromotion at major regions of the bone-implant interface.

## Data Availability

The data presented in this study are available on request from the corresponding author. The data are not publicly available due to their large size and unavailability of storage.

## Supplementary Materials

The following are available online at https://www.mdpi.com/1996-1944/14/1/114/s1, Figure S1: 3D re-entrant structure and the considered joints for analytical solution, Figure S2: Forces and moments required to cause (a) lateral displacement with no rotation, (b) pure axial extension, Table S1: Geometrical and material (Bernoulli or Timoshenko) parameters used in the analytical solution.
