# Supplementary material for: Femur Auxetic Meta-Implants with Tuned Micromotion Distribution
Source: Materials (Basel). 2020 Dec 29;14(1):114. doi: 10.3390/ma14010114 (PMC7795950; doi:10.3390/ma14010114)
Supplement: Supplementary file 1 [file materials-14-00114-s001.pdf]

Supplementary material

Femur Auxetic Meta-Implants With Tuned Micromotion Distribution

Naeim Ghavidelnia [1], Mahdi Bodaghi [2] and Reza Hedayati [3,*]

In this supplementary, we present the details of mathematical calculations used for obtaining the elements of the stiffness matrix of the 3D re-entrant unit cell. The analytical approach used in this study to obtain the mechanical properties of the structure was the same approach which we have previously introduced and implemented for obtaining analytical solutions for other structures such as rhombic dodecahedron [1], truncated cube [2], octahedral [3], rhombicuboctahedron [4], etc. As mentioned in the main paper, to calculate the analytical relationships for mechanical properties of the 3D re-entrant structure in the x and y directions, due to several loading and geometrical symmetries, 12 distinct degrees of freedom (DOFs) were considered (Figure S1) to define the behavior of the unit cell in response to two main loading conditions (i.e. F_x and F_y). Seven degrees of freedom q_1, q_2, q_3, q_4, q_5, q_6 and q_{12} have been considered to obtain the mechanical properties of the unit cell under F_y loading, and five other DOFs have been added to calculate the mechanical properties due to F_x loading condition. The deformations of the struts during the loading conditions could be considered as longitudinal and lateral deformations as shown in Figure S2. The parameters S_i and T_i are provided for several types of struts in Table S1 for both Euler-Bernoulli and Timoshenko beam theories. In the rest of this document, the procedure of obtaining elements of the stiffness matrix for the seven DOFs considered for F_y loading condition is presented in details, and the remaining procedures related to the other five DOFs are not presented in this document for the sake of brevity and also due to similarity. Moreover, since the stiffness matrix of 3D re-entrant unit cell is a symmetric matrix due to the geometrical and loading conditions ($K_{ij} = K_{ji}$), the procedure of obtaining the elements of the upper triangular section of the stiffness matrix are presented only. In other words, the procedure of obtaining the elements of K_{ij} with $i > j$ are not presented in this document for the sake of brevity.

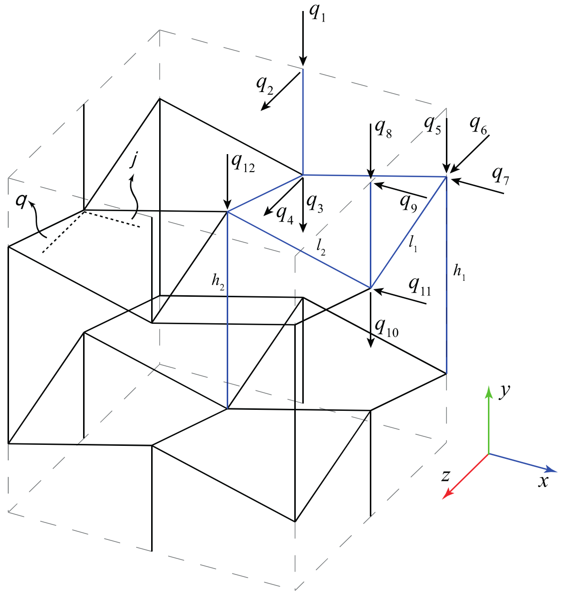

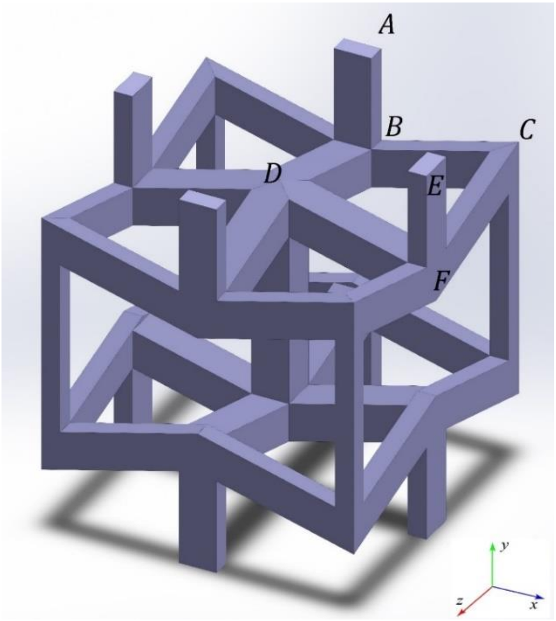

Copyright: © 2020 by the authors. Licensee MDPI, Basel, Switzerland. This article is an open access article distributed under the terms and conditions of the Creative Commons Attribution (CC BY) license (http://creativecommons.org/licenses/by/4.0/).

Figure S1. 3D re-entrant structure and the considered joints for analytical solution.

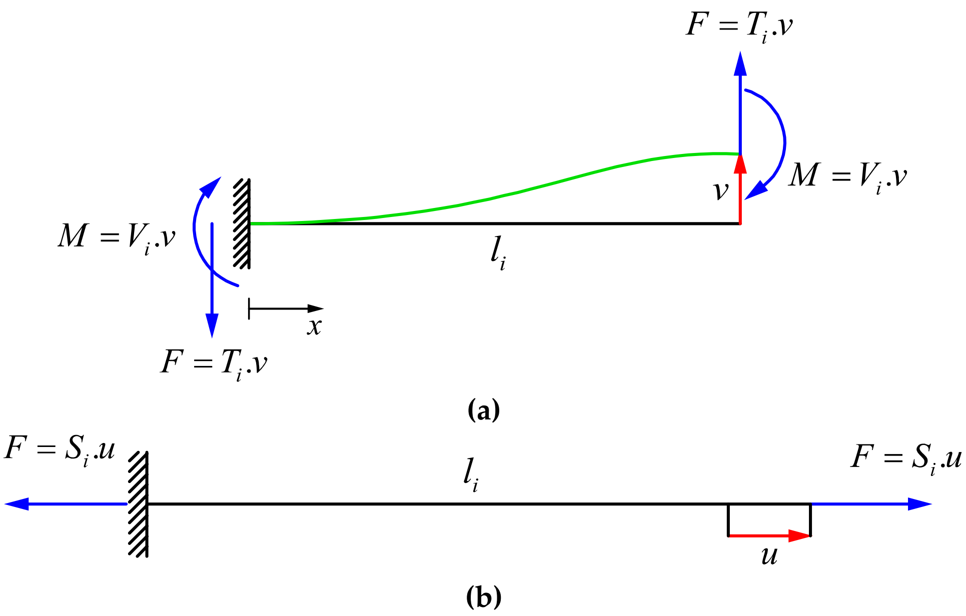

Figure S2. Forces and moments required to cause (**a**) lateral displacement $\boldsymbol{\delta}$ with no rotation, (**b**) pure axial extension

.**Table S1.** Geometrical and material (Bernoulli or Timoshenko) parameters used in the analytical solution.

Geometrical parameters	
$A_1 = \frac{t^2}{4}$	$I_1 = \frac{t^4}{192}$
$A_2 = \frac{t^2}{2}$	$I_{21} = \frac{t^4}{96}$
$A_3 = t^2$	$I_{22} = \frac{t^4}{24}$
$I_3 = \frac{t^4}{12}$	
Longitudinal parameters of both Euler-Bernoulli and Timoshenko beam theories	
$S_1 = \frac{t^2 E}{4H_1}$	$S_2 = \frac{t^2 E}{2H_1}$
$S_3 = \frac{t^2 E}{2l_1}$	$S_4 = \frac{t^2 E}{H_2}$
$S_5 = \frac{t^2 E}{l_2}$	
Lateral parameters of Euler-Bernoulli (left) and Timoshenko (right) beam theories	
$T_1 = \frac{t^4 E}{2H_1^3}$	$T_1 = \frac{4t^4 E}{(H + L\mathrm{Tan}[\theta])(\frac{8t^2(1+v)}{k} + (H + L\mathrm{Tan}[\theta])^2)}$
$T_2 = \frac{t^4 E}{H_1^3}$	$T_2 = \frac{8t^4 E}{(H + L\mathrm{Tan}[\theta])(\frac{8t^2(1+v)}{k} + (H + L\mathrm{Tan}[\theta])^2)}$
$T_3 = \frac{t^4 E}{8l_1^3}$	$T_3 = \frac{kt^4 E\mathrm{Cos}[\theta]^3}{kL^3 + 2Lt^2(1+v)\mathrm{Cos}[\theta]^2}$
$T_4 = \frac{t^4 E}{2l_1^3}$	$T_4 = \frac{4kt^4 E\mathrm{Cos}[\theta]^3}{kL^3 + 8Lt^2(1+v)\mathrm{Cos}[\theta]^2}$

$T_5 = \frac{t^4 E}{l_2^3}$	$T_5 = \frac{8kt^4 E \text{Cos}[\phi]^3}{kL^3 + 8Lt^2(1+v)\text{Cos}[\phi]^2}$

a) First DOF: $\boldsymbol{q_1 = 1}$

This DOF only deforms strut AB in its longitudinal direction. The equations of equilibrium for this strut at points A and B are as follows:

$$\sum F_{y,A} = 0 \rightarrow -\frac{Q_1}{4} + 2S_2 = 0 \rightarrow K_{11} = 8S_2 \quad (1)$$

$$\sum F_{y,B} = 0 \rightarrow -\frac{Q_3}{4} - 2S_2 = 0 \rightarrow K_{13} = -8S_2 \quad (2)$$

b) Second DOF: $\boldsymbol{q_2 = 1}$

This DOF only deforms strut AB in its lateral direction. The equations of equilibrium for this strut at point A are as follows:

$$\sum F_{z,A} = 0 \rightarrow -\frac{Q_2}{4} + T_2 = 0 \rightarrow K_{22} = 4T_2 \quad (3)$$

$$\sum F_{z,B} = 0 \rightarrow -\frac{Q_4}{4} - T_2 = 0 \rightarrow K_{24} = -4T_2 \quad (4)$$

c) Third DOF: $\boldsymbol{q_3 = 1}$

This DOF deforms struts AB, BC, and BD in their longitudinal and lateral directions. The equations of equilibrium for this struts at point B, C, and D are as follows:

$$\sum F_{y,B} = 0 \rightarrow -\frac{Q_3}{4} + 2S_2 + 2T_4\text{Cos}[\theta]^2 + 2S_3\text{Sin}[\theta]^2 + T_5\text{Cos}[\phi]^2 + S_5\text{Sin}[\phi]^2 = 0 \rightarrow K_{33} = 4(2S_2 + 2\text{Sin}[\theta]^2 S_3 + \text{Sin}[\phi]^2 S_5 + 2\text{Cos}[\theta]^2 T_4 + \text{Cos}[\phi]^2 T_5) \quad (5)$$

$$\sum F_{z,B} = 0 \rightarrow -\frac{Q_4}{4} - S_5\text{Sin}[\phi]\text{Cos}[\phi] + T_5\text{Sin}[\phi]\text{Cos}[\phi] = 0 \rightarrow K_{34} = 4\text{Cos}[\phi]\text{Sin}[\phi](-S_5 + T_5) \quad (6)$$

$$\sum F_{y,C} = 0 \rightarrow -\frac{Q_5}{8} - T_4\text{Cos}[\theta]^2 - S_3\text{Sin}[\theta]^2 = 0 \rightarrow K_{35} = -8(\text{Sin}[\theta]^2 S_3 + \text{Cos}[\theta]^2 T_4) \quad (7)$$

$$\sum F_{z,C} = 0 \rightarrow -\frac{Q_7}{8} + T_4\text{Cos}[\theta]\text{Sin}[\theta] - S_3\text{Sin}[\theta]\text{Cos}[\theta] = 0 \rightarrow K_{37} = 8\text{Cos}[\theta]\text{Sin}[\theta](-S_3 + T_4) \quad (8)$$

$$\sum F_{y,D} = 0 \rightarrow -\frac{Q_{12}}{2} - 2S_5\text{Sin}[\phi]^2 - 2T_5\text{Cos}[\phi]^2 = 0 \rightarrow K_{312} = -4(\text{Sin}[\phi]^2 S_5 + \text{Cos}[\phi]^2 T_5) \quad (9)$$

d) Forth DOF: $\boldsymbol{q_4 = 1}$

This DOF deforms struts AB, BC, and BD in their longitudinal and lateral directions. The equations of equilibrium for this struts at point B, C, and D are as follows:

$$\sum F_{z,B} = 0 \rightarrow -\frac{Q_4}{4} + T_2 + 2T_3 + S_5\text{Cos}[\phi]\text{^}2 + T_5\text{Sin}[\phi]\text{^}2 = 0 \rightarrow K_{44} = 4(\text{Cos}[\phi]^2 S_5 + T_2 + 2T_3 + \text{Sin}[\phi]^2 T_5) \quad (10)$$

$$\sum F_{z,C} = 0 \rightarrow -\frac{Q_6}{8} - T_3 = 0 \rightarrow K_{46} = -8T_3 \quad (11)$$

$$\sum F_{y,D} = 0 \rightarrow -\frac{Q_{12}}{2} + 2S_5\text{Sin}[\phi]\text{Cos}[\phi] - 2T_5\text{Sin}[\phi]\text{Cos}[\phi] = 0 \rightarrow K_{412} = 2\text{Sin}[2\phi](S_5 - T_5) \quad (12)$$

e) Fifth DOF: $\boldsymbol{q_5 = 1}$

This DOF deforms struts BC, CC, and CF in their longitudinal and lateral directions. The equations of equilibrium for this struts at point B, C, and F are as follows:

$$\sum F_{y,C} = 0 \rightarrow -\frac{Q_5}{8} + 2S_1 + 2T_4\text{Cos}[\theta]^\wedge 2 + 2S_3\text{Sin}[\theta]^\wedge 2 = 0 \rightarrow K_{55} = 16(S_1 + \text{Sin}[\theta]^2 S_3 + \text{Cos}[\theta]^2 T_4) \tag{13}$$

$$\sum F_{z,C} = 0 \rightarrow -\frac{Q_6}{8} + S_3\text{Sin}[\theta]\text{Cos}[\theta] - T_4\text{Sin}[\theta]\text{Cos}[\theta] = 0 \rightarrow K_{56} = 4\text{Sin}[2\theta](S_3 - T_4) \tag{14}$$

$$\sum F_{x,C} = 0 \rightarrow -\frac{Q_7}{8} + S_3\text{Sin}[\theta]\text{Cos}[\theta] - T_4\text{Sin}[\theta]\text{Cos}[\theta] = 0 \rightarrow K_{56} = 4\text{Sin}[2\theta](S_3 - T_4) \tag{15}$$

$$\sum F_{y,F} = 0 \rightarrow -\frac{Q_{10}}{4} - 2T_4\text{Cos}[\theta]^2 - 2S_3\text{Sin}[\theta]^2 = 0 \rightarrow K_{510} = -8(\text{Sin}[\theta]^2 S_3 + \text{Cos}[\theta]^2 T_4) \tag{16}$$

f) Sixth DOF: $\boldsymbol{q_6} = \mathbf{1}$

This DOF deforms struts BC and CF in their longitudinal and lateral directions. The equations of equilibrium for this struts at points C and F are as follows:

$$\sum F_{z,C} = 0 \rightarrow -\frac{Q_6}{8} + T_3 + S_3\text{Cos}[\theta]^\wedge 2 + T_4\text{Sin}[\theta]^\wedge 2 = 0 \rightarrow K_{66} = 8(\text{Cos}[\theta]^2 S_3 + T_3 + \text{Sin}[\theta]^2 T_4) \tag{17}$$

$$\sum F_{y,F} = 0 \rightarrow -\frac{Q_{10}}{4} + 2T_4\text{Cos}[\theta]\text{Sin}[\theta] - 2S_3\text{Sin}[\theta]\text{Cos}[\theta] = 0 \rightarrow K_{610} = 8\text{Cos}[\theta]\text{Sin}[\theta](-S_3 + T_4) \tag{18}$$

g) Twelfth DOF: $\boldsymbol{q_{12}} = \mathbf{1}$

This DOF deforms struts DD, DB, and DF in their longitudinal and lateral directions. The equations of equilibrium for this struts at point D is as follows:

$$\sum F_{z,C} = 0 \rightarrow -\frac{Q_{1212}}{2} + 4S_5\text{Sin}[\phi]^\wedge 2 + 4T_5\text{Cos}[\phi]^\wedge 2 + 2S_4 = 0 \rightarrow K_{1212} = 4(S_4 + 2\text{Sin}[\theta]^2 S_5 + 2\text{Cos}[\theta]^2 T_5) \tag{19}$$

References

1. Ghavidelnia, N.; Hedayati, R.; Sadighi, M.; Mohammadi-Aghdam, M. Development of porous implants with non-uniform mechanical properties distribution based on CT images. *Appl. Math. Model.* **2020**, *83*, 801–823, doi:10.1016/j.apm.2020.03.002.
2. Hedayati, R.; Sadighi, M.; Mohammadi-Aghdam, M.; Zadpoor, A.A. Mechanical properties of regular porous biomaterials made from truncated cube repeating unit cells: Analytical solutions and computational models. *Mater. Sci. Eng. C* **2016**, *60*, 163–183, doi:10.1016/j.msec.2015.11.001.
3. Hedayati, R.; Sadighi, M.; Aghdam, M.; Zadpoor, A. Analytical relationships for the mechanical properties of additively manufactured porous biomaterials based on octahedral unit cells. *Appl. Math. Model.* **2017**, *46*, 408–422, doi:10.1016/j.apm.2017.01.076.
4. Hedayati, R.; Sadighi, M.; Mohammadi-Aghdam, M.; Zadpoor, A. Mechanics of additively manufactured porous biomaterials based on the rhombicuboctahedron unit cell. *J. Mech. Behav. Biomed. Mater.* **2016**, *53*, 272–294, doi:10.1016/j.jmbbm.2015.07.013.
